# On Fatty Degeneration of the Arteries, with a Note on Some Other Fatty Degenerations

**Published:** 1843-06

**Authors:** George Gulliver


*On Fatty Degeneration of the Arteries, with a Note on some other Fatty Degenerations.* By George Gulliver, F. R. S.—The author, remarking how vaguely the epithets, atheromatus, steatomatous, &c., have been applied by pathological writers to diseased arteries, and that the morbid deposit between the middle and inner coats, and in the substance of the former, has not, as far as he knows, yet been submitted to precise examination, gives the result of his own observations, from which it appears that the disease is really of a fatty nature. A microscopic examination of it brings into view a multitude of crystalline plates, fatty globules, with albuminous and earthy particles. Several specimens of the crystals were sent for examination to Dr. Davy, who ascertained that they are of cholesterine.

The fatty matter is easily extracted by boiling alcohol, and the chrystals of cholesterine are seen to be deposited as the solution cools. The author has examined numerous specimens of the disease, and never failed to observe these crystals and the fatty globules in the deposit, and also generally in the substance of the altered middle coat. The microscopic characters are given in two figures.

The accuracy of Dr. Davy's observations (see his "Researches, Phys. and Anat.," vol. I., pp. 372 and 436) as to the thinning, &c., of the middle coat of the artery, is confirmed by Mr. Gulliver.

The importance of fatty degeneration of the coats of the arteries is insisted on, especially as to its general connection with thickening and puckering of the inner membrane, with aneurism, with obstruction, occlusion, or ossification of the vessels, and of those ruptures of them which are so frequently the cause of sudden death.

The author adds, that fatty degenerations are more common and of more importance than has yet been supposed. He mentions obstruction, by fatty particles, of the seminal tubes; and notices fatty degenerations of the blood, lungs, &c. The disease he describes as being more remarkable in "brown consolidation" of the lungs than in red consolidation; and these two diseases are described as affording distinct morbid products.—*Med. Ex.*, from *Provincial Med. Journ.*, March 18, 1843.

